# Comparison of single-injection ultrasound-guided thoracic paravertebral block with transversus abdominis plane block in peritoneal dialysis catheter implantation: a randomized controlled trial

**DOI:** 10.1186/s13063-021-05223-7

**Published:** 2021-04-09

**Authors:** Xiao-juan Jiang, Zi Li, Qi Li, Hai-yan Zhang, Xiao-hong Tang, Tao Zhu

**Affiliations:** 1 Department of Anesthesiology, West China Hospital, Sichuan University & The Research Units of West China (2018RU012), Chinese Academy of Medical Sciences, Chengdu, 610041 Sichuan China; 2 Department of Nephrology, West China Hospital, Sichuan University & The Research Units of West China (2018RU012), Chinese Academy of Medical Sciences, Chengdu, 610041 Sichuan China

**Keywords:** Peritoneal dialysis, Kidney failure, chronic, Thoracic paravertebral block, Transversus abdominis plane block, Regional anesthesia, Ultrasonography

## Abstract

**Background:**

Previous study indicated that transversus abdominis plane (TAP) block could be the principal anesthetic technique for *p*eritoneal dialysis catheter (PDC) implantations*.* However, a TAP block could not provide an optimal anesthetic effect on catheter exit site during PDC implantation. We hypothesized that single-injection ultrasound-guided thoracic paravertebral block (US-TPVB) could be the principal anesthetic technique with better pain relief at catheter exit site during PDC implantation, compared to a TAP block. And anesthesia quality of a single-injection US-TPVB was compared with that of a TAP block and local anesthetic infiltration (LAI)*.*

**Methods:**

Patients undergoing PDC implantations were randomized into groups TPVB or TAP or LAI. In group TPVB, single-injection US-TPVB at T10-T11 level was performed with 20 ml of 0.25% ropivacaine. In group TAP, oblique subcostal TAP block was performed with 20 ml of 0.25% ropivacaine. In group LAI, 40 ml of 0.25% ropivacaine was used. Anesthesia quality was compared among the three groups, including general anesthesia conversion rate, cumulative rescuing sufentanil consumption, and satisfaction rate by nephrologists and patients.

**Results:**

Eighty-eight eligible patients were enrolled. Visual analogue scale (VAS) at most time points (except for the catheter exit site) were lower in group TAP, compared with group TPVB. VAS at parietal peritoneum manipulation was 6 (5, 7), 3 (0, 6), and 7 (4.75, 9) in groups TPVB, TAP, and LAI, respectively (*P* < 0.001). VAS at catheter exit site was 4 (3, 4), 5.5 (4, 8), and 5 (3, 7.25) in groups TPVB, TAP, and LAI, respectively (*P =* 0.005). Lower general anesthesia conversion rate, less cumulative rescuing sufentanil consumption, and higher satisfaction rates by nephrologists and patients were recorded in group TAP, compared with groups TPVB and LAI.

**Conclusions:**

Single-injection US-TPVB provided a better pain relief at catheter exit site. The quality and reliability of anesthesia after a single-injection US-TPVB was comparable to that of LAI, but not better than that of an oblique subcostal TAP block for PDC implantation.

**Trial registration:**

TCTR20160911002. Registered on 8 September 2016.

## Background

In developing countries, open dissection peritoneal dialysis catheter (PDC) implantation is still a common procedure for renal replacement for patients with end-stage renal diseases (ESRD) [1]. Co-existing cardiopulmonary comorbidities and coagulopathy in ESRD patients contribute that neither general anesthesia (GA) nor neuraxial anesthesia is a proper anesthetic solution for those patients [2, 3]. Therefore, local anesthetic infiltration (LAI) is still a common anesthetic option for PDC implantation in ESRD patients. However, LAI cannot provide good anesthesia for peritoneum stimulation, which results in visceral pain during PDC implantation. Great efforts have been put to explore a better anesthetic solution for PDC implantation [4, 5].

In our previous studies, it was demonstrated that ultrasound-guided oblique subcostal transversus abdominis plane (TAP) block could be the principal anesthetic technique for PDC implantations and produced better anesthesia than LAI did, especially on peritoneum stimulation [4, 5]. However, the catheter exit site could not be covered by a TAP block. The catheter exit site was innervated by both the lateral and anterior cutaneous branches of related somatic nerve and a TAP block could block the anterior cutaneous branches only [6].

In recent decade, as a novel regional anesthetic technique, ultrasound-guided thoracic paravertebral block (US-TPVB) has been more and more extensively used. Usually, TPVB is performed for postoperative analgesia in thoracic and abdominal surgeries [7–9]. Anatomic study has indicated that TPVB could block ipsilateral, segmental, somatic, and sympathetic nerve, including both the lateral and anterior cutaneous branches of the related somatic nerve. Therefore, TPVB could possibly provide anesthesia not only in the skin and muscles of the related unilateral chest and abdominal wall, but also partially the parietal peritoneum attributed to unilateral sympathetic block [10]. In our recently published study, it was demonstrated that a single-injection US-TPVB could be the principal anesthetic technique for PDC procedures and provided a comparable anesthetic effect to that of LAI [11].

We hypothesize that a single-injection US-TPVB could be the principal anesthetic technique with a better pain relief at catheter exit site, compared to a TAP block. And anesthetic quality of a single-injection US-TPVB was compared with that of a TAP block and LAI.

## Methods

This study was registered at the TCTR (http:// www.clinicaltrials.in.th, registration number is TCTR20160911002) and approved by the Ethics Committee of West China Hospital of Sichuan University (approval number: 2017-267) before the first patient was enrolled. The study was conducted in accordance with the Declaration of Helsinki, and written informed consent was obtained from all participants.

**Fig. 1:**
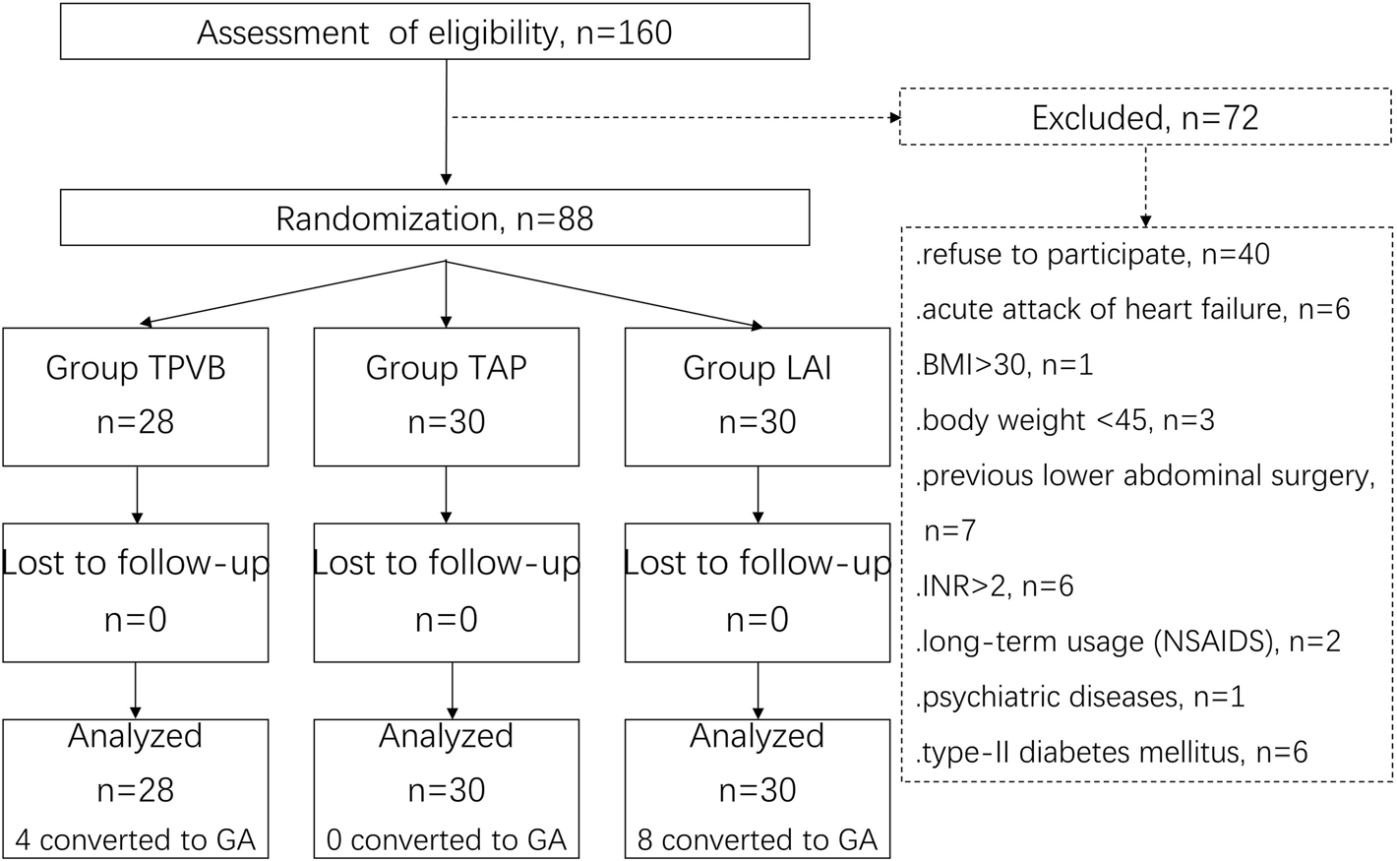
The research flowchart. TPVB, thoracic paravertebral block; TAP, transversus abdominis plane; LAI, local anesthetic infiltration; GA, general anesthesia

ESRD patients undergoing PDC implantations and preferred regional anesthesia (TPVB, TAP or LAI) to general anesthesia in West China Hospital were invited to participate in this study (Fig. 1). Individuals were eligible if they were 18–75 years old and had no contraindication for PDC implantation. Exclusion criteria were patients who had any anatomical abnormalities or infections in the spine or paravertebral region, uncontrolled severe acute heart failure (New York Heart Association, NYHA Grade III-IV), severe obesity [body mass index (BMI) > 30], body weight < 45 kg, severe coagulopathy (international normalized ratio [INR] > 2), previous low abdominal surgery, allergy to ropivacaine, long-term usage (over 3 consecutive months) of opiates or non-steroidal anti-inflammatory drugs (NSAIDS), cerebrovascular or psychiatric diseases or poor consciousness level or type II diabetes mellitus with peripheral neuropathy preventing realistic sensation to pain, inability to communicate, and pregnancy. Patient enrollment was done by investigators 2 and 5 (L.Z. and T.X.H.). A research assistant generated the random allocation sequence (using computer-generated random numbers) and put the allocation sequence in sealed opaque sequentially numbered envelopes. On the day of surgery in the block room, investigator 3 (L.Q.) opened one envelope per patient and performed all regional (real and sham) blocks according to the allocation of the patient. Then, investigator 3 had no further involvement in this study until completion of data analysis. All eligible patients were randomized into groups TPVB or TAP or LAI. All other investigators and patients were blinded to group allocation. Blinding was not revealed until statistical analysis was completed if no severe adverse event occurred during the study.

All patients were on NPO (nothing by mouth) preoperatively and got standard anesthesia monitoring (electrocardiogram [ECG], blood oxygen saturation [SPO_2_], non-invasive blood pressure [NIBP]) in a block room. Intravenous sufentanil 2.5 μg and midazolam 1 mg were given for sedation. All patients received a similar anesthetic protocol: all patients were laid in a lateral decubitus position with the operative side on top at first; after the real or sham US-TPVB was performed, patients were changed to supine position for the real or sham TAP block and LAI. The nephrologists marked sites of incision, subcutaneous tunnel, and catheter exit in advance. 0.25% ropivacaine (diluted with Naropin (1% ropivacaine), Astra Zeneca, London, UK) was used for regional blocks.

An ultrasound machine (M-Turbo, SonoSite Inc., Seattle, WA, USA) with a low-frequency (2–5 MHz) curved probe was used for TPVB and a linear high frequency probe (6–13 MHz) was used for TAP block. The probes were covered with a sterile, single-use, ultrasound probe sheath. A 22-gauge, 100-mm-long, short oblique needle (Braun, Germany) was used for regional blocks. For US-TPVB, the target thoracic vertebral level (T10-T11) was identified by palpating and counting down from vertebra prominens (C7). After skin disinfection and sterile preparation, a single-injection US-TPVB was performed in an intercostal approach at the level between T10 and T11, as that described in Taketa’s paper [12]. Ultrasound-guided oblique subcostal TAP block described by Hebbard was performed only in group TAP [6].

In group TPVB, the real US-TPVB was conducted with 20 ml of 0.25% ropivacaine on the operative side. Then, the sham TAP was subcutaneous injection of 1 ml normal saline and the sham LAI was injection of 10 ml of normal saline at the incision and the catheter exit site. In group TAP, the sham US-TPVB was performed with subcutaneous injection of 1 ml normal saline. The real TAP was performed with 20 ml of 0.25% ropivacaine on the operative side and the sham LAI was injection of 10 ml of normal saline at the incision and the catheter exit site. In group LAI, the sham US-TPVB and TAP were performed using subcutaneous injections of 1 ml of normal saline on operative side, respectively. Then, the real LAI was performed by investigator 3 with subcutaneous injections 40 ml of 0.25% ropivacaine at the incision, the subcutaneous tunnel, and the catheter exit site.

Fifteen minutes after ropivacaine injection, sensation loss was tested using pinprick. The area with surgical anesthesia was defined as the area where the visual analogue scale (VAS) of pain was less than 2, tested using pinprick without any rescuing sufentanil administration. Preoperative VAS evaluation was performed by investigator 3 via the method described in Ferreira-Valente’s study [13]. Then, the patient was moved into an operating room. The surgery was started 30 min after regional anesthesia was completed. All patients received double-cuff, straight-tip Tenckhoff catheters, and prophylactic antibiotics were given preoperatively. All PDC implantations with open dissection were done as per Markic’s study [14].

Intraoperative and postoperative pain were evaluated using VAS from 0 to 100 mm (0 is no pain and 100 is worst imaginable pain) by investigators 1 (J.X.J) and 4 (Z.H.Y) according to the method in Ferreira-Valente’s study [13]. As a rescue regime, sufentanil 2.5 μg was given when the VAS was over 5, with the total rescuing dose limited to 7.5 μg. Anesthesia was converted to general anesthesia when the VAS was still over 5 after a total dose of 7.5 μg rescue sufentanil had been given. Patients converted to GA were not included in pain analyses. For general anesthesia, sufentanil 0.15 μg/kg, midazolam 2 mg, propofol 1.5 mg/kg, and cisatracurium 0.2 mg/kg were given for anesthesia induction, followed by an LMA (Teleflex Medical Europe Ltd., Athlone, Ireland) insertion. General anesthesia was maintained with continuous infusion of propofol (5 mg/kg/h) and remifentanil (0.1 μg/kg/min). A 1000 mL 20% long-chain triglyceride emulsion (Intralipid, Fresenius Kabi, Uppsala, Sweden) was prepared for local anesthetic systemic toxicity (LAST) management. Patients undergoing GA were moved to a post-anesthesia care unit after surgery and received standard monitoring until they reached the discharge standard. Postoperative rescuing analgesic regimen was intramuscular tramadol 100 mg whenever the VAS was above 5, and a total dose was limited to 400 mg within 24 h.

### Outcome measurement

The primary outcomes were VAS scores at parietal peritoneum manipulation (parietal peritoneum purse-string sutures) and catheter exit site. VAS was evaluated at the following time points: skin incision, division of subcutaneous tissues, parietal peritoneum manipulation, catheter exit, incision closing, at rest, and with coughing at 2 and 24 postoperative hours.

Cumulative rescue sufentanil dosage and conversion rate from regional anesthesia to GA were compared among the three groups. Anesthesia quality was assessed by the nephrologist and patients respectively using a 4-point rating scale (excellent, good, poor, bad) immediately after surgery was completed. The nephrologist rated anesthesia quality according to laxity of the rectus abdominis and activity of the great omentum. Anesthesia quality rating of “excellent” and “good” were regarded as infer “satisfaction with technique” and quality rating of “poor” and “bad” were taken to “dissatisfaction with technique.”

Any surgical or anesthetic complications occurring within 4 weeks of implantation were recorded.

### Statistical analysis

Sample size calculation was based on previous study [4, 11]. The hypothesis was that TPVB would reduce patient VAS scores by 40% at catheter exit site, compared to TAP block. Using one-way ANOVA test at the 5% level of significance, a sample of 22 patients per group would yield 80% power to detect a difference of this magnitude. Considering possible subjects lost in 4 postoperative weeks, other 22 patients (one third of 66) were enrolled, a total of 88. The Shapiro-Wilk test was performed to determine the normal distribution of continuous variables. Two separate one-way ANOVAs (with the independent two-sample Student’s t test for post hoc testing) were used in analyzing continuous data. Non-parametric data were presented as median values with their interval from the 25th to 75th percentile and were tested with independent-samples Kruskal-Wallis test. The Fisher exact test (with the Mann-Whitney *U* test for post hoc testing) was used in analyzing categorical data. For repeated outcome measurements, the *P* values were corrected using the Bonferroni-Holm adjustment. A *P* value less than 0.017 (0.05/3) was regarded as statistically significant in independent-samples Kruskal-Wallis test for the two primary outcomes. The data were analyzed using SPSS 19.0 for Windows (SPSS Inc., Chicago, IL, USA).

## Results

From 1 July 2018 to 30 June 2019, 160 patients were invited and 72 patients were excluded for either not meeting the inclusion criteria (*n* = 32) or refusing to participate (*n* = 40), and eighty-eight eligible patients were included and randomized into three groups (Fig. 1). There were no differences with regard to age, gender ratio, body weight, cardiopulmonary co-existing diseases, or ASA physical status among the three groups (Table 1).

**Table 1:** Demographic data

Characteristic	Group TPVB *n* = 28	Group TAP *n* = 30	Group LAI *n* = 30	*P*
Male, *n* (%)	13 (46.4)	12 (40)	13 (43.3%)	0.56
Age (years)	43.0 ± 12.6	43.3 ± 12.7	41.2 ± 12.6	0.94
Body weight (kg)	57.1 ± 10.3	57.6 ± 8.9	60.2 ± 11.0	0.88
BMI	21.5 ± 3.1	21.5 ± 2.8	22.0 ± 1.4	0.91
ASA physical status: III/IV	25 (89.3)/3 (10.7)	24 (80)/6 (20)	25 (83.3)/5 (16.7)	0.62
Renal hypertension	22 (78.6)	25 (83.3)	26 (86.7)	0.71
Pulmonary infection or COPD	2 (7.1)	4 (13.3)	5 (16.7)	0.54
Cardiac insufficiency (NYHA classic: grade III/IV)	5 (17.9)	7 (23.3)	6 (20)	0.87
Causes of chronic renal insufficiency				0.72
Chronic nephritis	4 (14.3)	7 (23.3)	8 (26.7)	
Hypertension	6 (21.4)	7 (23.3)	9 (30.0)	
Diabetic nephropathy	2 (7.1)	3 (10.0)	1 (3.3)	
Obstructive kidney disease	2 (7.1)	1 (3.3)	0 (0)	
Unknown	10 (35.7)	10 (33.3)	11 (36.7)	
Others	4 (14.3)*	2 (6.7)^#^	1 (3.3)^+^	

Data were presented as mean ± SD, or number (%). *P* values were calculated using one-way ANOVA test (with the independent two-sample Student’s *t* test for post hoc testing) or Fisher’s exact test (with the Mann-Whitney *U* test for post hoc testing), as appropriate

*TPVB* thoracic paravertebral block, *TAP* transversus abdominis plane, *LAI* local anesthesia infiltration

*Three cases of gouty nephropathy and one case of polycystic kidney disease. ^#^One case of ANCA (anti-neutrophil cytoplasmic antibodies) associated vasculitis and one case of allergic purpura nephritis. ^+^One case of hepatitis B virus-associated glomerulone nephritis

### VAS scores of pain

VAS was significantly lower in group TAP in most of the time points (except for catheter exit site and 24 h postoperatively), compared to group TPVB. VAS was comparable between groups TPVB and LAI in most of the time points (Table 2).

**Table 2:** Perioperative visual analogue scale of pain

Time points	Group TPVB *n* = 28	Group TAP *n* = 30	Group LAI *n* = 30	*P*
Skin incision	5 (3, 7)^#^	3 (1, 5)*	3 (1.75, 5)*	0.002
Division of subcutaneous tissue	4 (2, 6)	2 (0, 3.25)*	2 (0.75, 4)	0.007
Parietal peritoneum manipulation	6 (5, 7)	3 (0, 6)*^,#^	7 (4.75, 9)	< 0.001
Catheter exit	4 (3, 4)	5.5 (4, 8)*	5 (3, 7.25)	0.005
Incision closing	4 (3, 5)	2 (0, 3)*	3 (0, 5)	0.004
At rest at 2 h postoperatively	2 (1, 3)^#^	1 (0, 2)*	1 (0, 1.25)	0.001
With coughing at 2 h postoperatively	2 (2, 3)	1 (0, 2)*^,#^	2 (2, 3)	< 0.001
At rest at 24 h postoperatively	1 (0, 2)	1 (0, 2)	1 (0, 1)	0.717
With coughing at 24 h postoperatively	2 (0, 3)	1 (0, 2)	2 (2, 2.25)	0.240

Data were presented as median (25th–75th percentile). *P* values were calculated using independent-samples Kruskal-Wallis test. Significance values have been adjusted by the Bonferroni correction for multiple tests. *Median *P* < 0.017, compared with groups TPVB. ^#^Median *P* < 0.017, compared with groups LAI

*TPVB* thoracic paravertebral block, *TAP* transversus abdominis plane, *LAI* local anesthetic infiltration

### Conversion rate to general anesthesia, anesthesia quality, and consumption of rescue analgesics

Four patients in group TPVB and eight in group LAI were converted to general anesthesia due to “bad” anesthesia after a total of 7.5 μg rescuing sufentanil. No patient in group TAP was converted to GA. Significantly lower GA conversion rate was recorded in group TAP, compared with that in groups TPVB and LAI (Table 3). There was no difference of GA conversion rate between groups TPVB and LAI.

**Table 3:** Parameters of anesthesia and surgery in three groups

Characteristic	Group TPVB *n* = 28	Group TAP *n* = 30	Group LAI *n* = 30	*P*
Ropivacaine/body weight (mg/kg)	0.90 ± 0.18	0.89 ± 0.14	1.41 ± 0.32	0.04
Switching into GA	4 (14.3)	0 (0)*^,#^	8 (26.7)	0.01
Surgery time (minutes)	48.9 ± 12.2	49 ± 14.6	58 ± 12.4	0.98
Regional block time (minutes)	7.57 ± 4.73	7.16 ± 3.42	6.7 ± 3.2	0.75
Quality of anesthesia rated by nephrologists				< 0.01
Satisfied—excellent/good	18 (64.3)—3 (10.7)/15 (53.6)	27 (90)*^,#^—17 (56.7)/10 (33.3)	14 (46.7)—6 (20)/8 (26.7)	
Dissatisfied—poor/bad	10 (35.7)—6 (21.4)/4 (14.3)	3 (10)*^,#^—3 (10)/0 (0)	16 (53.2)—8 (26.6)/8 (26.6)	
Quality of anesthesia rated by patients				< 0.01
Satisfied—excellent/good	18 (64.3)—3 (10.7)/15 (53.6)	27 (90)*^,#^—17 (56.7)/10 (33.3)	16 (53.3)—1 (3.3)/15 (50)	
Dissatisfied—poor/bad	10 (35.7)—6 (21.4)/4 (14.3)	3 (10)*^,#^—3 (10)/0 (0)	14 (46.6)—6 (20)/8 (26.6)	
Cumulative rescuing sufentanil (μg)	7.3 ± 2.9	5.3 ± 2.6*^,#^	6.5 ± 2.2	0.01

Data were presented as mean ± SD, or number (%). *P* values were calculated using one-way ANOVA test (with the independent two-sample Student’s *t* test for post hoc testing) or Fisher’s exact test (with the Mann-Whitney *U* test for post hoc testing), as appropriate. *Means *P* < 0.017, compared to group TPVB; ^#^*P* < 0.017, compared to group LAI

*TPVB* thoracic paravertebral block, *TAP* transversus abdominis plane

Significantly higher rates of satisfaction in nephrologists and patients with the anesthetic technique were observed in group TAP, compared with groups TPVB and LAI (Table 3). There was no difference of anesthesia satisfaction rate between groups TPVB and LAI.

### Boundaries of area with surgical anesthesia in groups TPVB and TAP

**Fig. 2:**
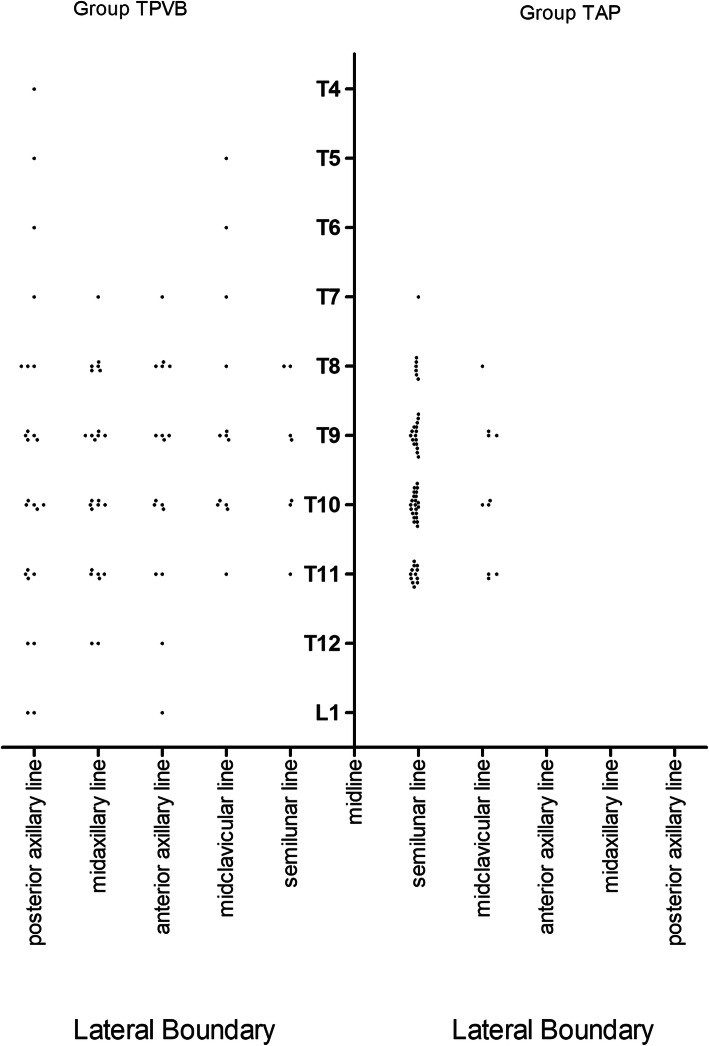
Boundaries of area with surgical anesthesia after a TPVB or TAP block. Note: each dot represented one patient whose lateral boundary of area with surgical anesthesia reaching to that line at that dermatome segment

In groups TPVB and TAP, dermatome segments of the upper, lower, and lateral boundaries with surgical anesthesia are shown in Table 4 and Fig. 2. There was a much greater variation of the size of area with surgical anesthesia in patients in group TPVB, compared to patients in group TAP (Fig. 2). The lateral boundary was beyond the ipsilateral semilunar line in the majority of patients in group TPVB. But in the majority of patients in group TAP, the lateral boundary was within the ipsilateral semilunar line. The medial boundary did not cross the midline in groups TPVB or TAP.

**Table 4:** Dermatome segments (upper and lower boundary) with surgical anesthesia tested with pinprick

Dermatome segment	Group TPVB *n* = 28	Group TAP *n* = 30
T4	1 (3.5)	0 (0)
T5	2 (7)	0 (0)
T6	2 (7)	0 (0)
T7	4 (14)	1 (3.3)
T8	15 (53.6)	7 (23.3)
T9	21 (75)	19 (63.3)
T10	21 (75)	25 (83.3)
T11	13 (46.4)	18 (60)
T12	5 (17.9)	0 (0)
L1	3 (10.7)	0 (0)

Values are number of patients with surgical anesthesia presented in that dermatome segment level (proportion of patients with surgical anesthesia presented in that dermatome segment level in the same group)

*TPVB* thoracic paravertebral block, *TAP* transversus abdominis plane, *T4–T12* thoracic nerves, *L1* lumbar nerves

### Surgical and anesthetic complications

There were no significant differences in surgical and anesthetic complications among the three groups in the 4-week follow-up. One catheter migration and one catheter obstruction were recorded in groups TAP and LAI. One case of dialysate leakage was recorded in group TPVB. Perioperative hemodynamics were stable. No LAST or other complications occurred.

## Discussion

Previous study had showed that parietal peritoneum manipulation and catheter exit site were the most uncomfortable part during PDC implantations for patients undergoing LAI or TAP block [4, 5]. Visceral pain during peritoneum manipulation was attributed to stimulation of autonomous (sympathetic and parasympathetic) nerve system innervating peritoneum. And poor pain relief at the catheter exit site in PDC implantations is because that catheter exit site is innervated by both the lateral and anterior cutaneous branches of the related somatic nerve and a TAP block blocks the anterior cutaneous branches only. Theoretically, TPVB might block ipsilateral, segmental, somatic, and sympathetic nerve. Consequently, TPVB would provide anesthesia not only in the skin and muscles of the related unilateral chest and abdominal wall, but also partially the parietal peritoneum due to unilateral sympathetic block [10]. Results in this study showed that, with adjunct of a small dose of rescuing sufentanil (a total of 7.5 μg), the majority of patients in group TPVB underwent PDC implantations successfully; similar result also was observed in our previous study [11]. And either TPVB or TAP block provided partial anesthetic effect on parietal peritoneum stimulation. A TPVB blocks both the lateral and anterior cutaneous branches of the related somatic nerves. Results in this study demonstrated TPVB provided a better pain relief at catheter exit site, compared to a TAP block. However, VAS at most of the other time-points (including peritoneum manipulation) during PDC implantation was significantly lower in group TAP, compared to groups TPVB.

In this study, the area with surgical anesthesia after a single-injection US-TPVB at T10–T11 level with 20 ml of 0.25% ropivacaine had an upper-lower boundary between T4 and L1, medial boundary not crossing the midline and lateral boundary reaching as far as to the ipsilateral posterior axillary line in some patients, which was much wider but more variable than that after a TAP block (see Fig. 2). Like results reported by Kotze et al. [15], the great variability of boundaries of area with surgical anesthesia after a single-injection TPVB in this study indicated poor predictability of spread of local anesthetics in a single-injection TPVB, which consequently affected the reliability of its block. In the contrast, a TAP block provided a more reliable and better anesthetic effect, though such an area with surgical anesthesia was smaller. This might be one of the reasons why lower VAS was observed at almost all time points during PDC implantations in group TAP. TAP blocks provided even a better anesthesia for parietal peritoneum stimulation than a TPVB did in this study. And lower GA conversion rate, higher “satisfied” anesthesia rates, and less rescuing sufentanil administration were recorded in group TAP, compared to groups TPVB and LAI. As regional anesthesia techniques, quality of both TPVB and TAP is highly dependent on volume of local anesthetics injected. To avoid LAST occurring in ESRD patients, 0.25% ropivacaine was used in this study. The low concentration of ropivacaine (0.25%) used in TPVB might be another factor which contributed to such a result in this study, but in fact the same concentration of ropivacaine was used in group TAP. A higher concentration, like 0.375% or 0.5%, with even reduced volume ropivacaine for TPVB should be tested in the future. Furthermore, a recent study showed that TAP combined with rectus sheath block can serve as the primary anesthetic modality for peritoneal dialysis catheter placement surgery [16]. For poor anesthesia effect after a TPVB, rectus sheath block might be another rescue solution. Therefore, as the principal anesthetic solution for PDC implantations, single-injection US-TPVB at T10-T11 level or LAI was not better than oblique subcostal TAP block.

There were several limitations in this study. First, we did not used any rescue local anesthetics during the procedure as that in a traditional way because we did not know the maximum safe dosage of local anesthetic in an ESRD patient after a TPVB or TAP block with 20 ml of 0.25% ropivacaine. Moreover, investigator 3 (L.Q.) performed all regional blocks and was not blinded to patient allocation, though he had no further involvement in the study until completion of data analysis.

## Conclusion

As the principal anesthetic technique in PDC implantations, single-injection US-TPVB provided a better pain relief at catheter exit site. The quality and reliability of anesthesia after a single-injection US-TPVB was comparable to that of LAI, but not better than that of an oblique subcostal TAP block for PDC implantation.

## Data Availability

The datasets used and/or analyzed during the current study are available from the corresponding author on reasonable request.

## Abbreviations

PDC: Peritoneal dialysis catheter
ESRD: End-stage renal diseases
LAI: Local anesthetic infiltration
TAP: Transversus abdominis plane
US-TPVB: Ultrasound-guided thoracic paravertebral block
GA: General anesthesia
VAS: Visual analog scale
LMA: Laryngeal mask airway
LAST: Local anesthetic systemic toxicity
